# *In Vivo* Antiplasmodial, Anti-Inflammatory, and Analgesic Properties, and Safety Profile of Root Extracts of *Haematostaphis barteri* Hook F. (Anacardiaceae)

**DOI:** 10.1155/2015/872892

**Published:** 2015-11-04

**Authors:** Johnson Nyarko Boampong

**Affiliations:** Department of Biomedical and Forensic Sciences, School of Biological Science, College of Agriculture and Natural Sciences, University of Cape Coast, Cape Coast, Ghana

## Abstract

Malaria is an endemic disease globally and the conundrum of drug resistance has led to the search for newer antimalarial agents. The root extract of* H. barteri* was evaluated for antimalarial, analgesic, and anti-inflammatory properties. The prophylactic effect of* H. barteri* on* P. berghei* was determined by pretreating mice with aqueous root extract of* H. barteri* (30–300 mg/kg), saline, and 1.2 mg/kg sulfadoxine/pyrimethamine for three days followed by 1 × 10^6^ *P. berghei* inoculation. Parasite density was measured after 72 h. The curative antimalarial property of the extract was assessed by treating mice with extract, saline, and 1.14 : 6.9 mg/kg Artemether : Lumefantrine four days after 1 × 10^6^ *P. berghei* inoculation. Selected organs were harvested for toxicity assessment. The anti-inflammatory and analgesic effect of the extract was determined in the carrageenan and thermal tail withdrawal tests, respectively. The extract significantly reduced the parasite density in the prophylactic but not the curative study. The anti-inflammatory and analgesic activities of the extract were significant (*P* < 0.05) only at the highest doses employed. Regeneration of hepatocytes was also evident in the extract treated groups. The extract has prophylactic but not curative activity on* P. berghei*-induced malaria. The anti-inflammatory and analgesic property of the extract occurred at the highest doses used.

## 1. Introduction

Malaria is a major global health threat and it affects more than 500 million people leading to annual mortality of 1–3 billion mainly among young children below 5 years. Malaria parasites produce diverse toxins which stimulate the host immune cells to produce excessive cytokines which adversely drive the disease progression [1]. Though the proinflammatory response protects host against the asexual blood stages of the parasite, it results in elevated temperature with its attendant chills and bodily pains. The inflammation in malaria is associated with elevated levels of proinflammatory cytokines, including interleukin 1*β* (IL-1*β*), IL-6, IL-8, IL-23, gamma interferon (IFN-*γ*), and tumor necrosis factor alpha [2–5]. On the other hand, decreased levels of additional proinflammatory cytokines, such as IL-12 and IFN-*α*, are associated with enhanced malaria pathogenesis in humans [6].

Artemisinin Combination Therapy (ACT) to some extent reduced the prevalence of the disease. The emerging multidrug resistance forms of the parasite, the resistant vector, use of substandard ACTs, and limited supply of the standard and affordable ACTs have intensified the reliance on the traditional herbal preparations by most people in the malaria endemic regions [7]. In Ghana, especially among Brifo and Wale communities in Upper West region, the boiled leaves of* Haematostaphis barteri* have been the mainstay of malaria treatment traditionally.* H. barteri* also known as blood plum is a member of Anacardiaceae family [8]. The stem bark is used to treat hepatitis while others take a decoction along with some other plants for the treatment of sleeping sickness. The roots of* H. barteri *are used in the treatment of swollen body parts [9]. It has been confirmed that the decoction of the leaves of* H. barteri* has antimalarial activity; the claim that the decoction of the root bark equally possesses antimalarial activity has not been scientifically proven.

This study therefore investigated the antimalarial, anti-inflammatory, and analgesic properties and safety profile of the root extract of the plant.

## 2. Materials and Methods

### 2.1. Collection and Identification of Plant Material

The roots of* Haematostaphis barteri* were collected from Wechiua in the Northern Region of Ghana in the months of December, 2012, and January, 2014, and authenticated by a botanist in the School of Biological Sciences. The roots were dried under shade for 14 days. A voucher specimen has been kept in the School of Biological Sciences' herbarium.

### 2.2. Preparation of Aqueous Root Extract of* Haematostaphis barteri*

The dried roots of* H. barteri* were pulverized with an electric mill. One hundred grams of the powdered roots was extracted with 300 mL of distilled water and maintained at 80°C for 24 h. The filtrate was evaporated and lyophilized by freeze drying.

### 2.3. Drugs and Chemicals

The drugs and chemicals used were sulfadoxine/pyrimethamine (SP) obtained from Maxheal Labs Pvt. Ltd. (Sachin, India), artemether and lumefantrine (A-L) were obtained from Ajanta Pharma Ltd. (Mumbai, India), and diclofenac sodium was purchased from Troge, Hamburg, Germany. Carrageenan, serotonin, and histamine were also purchased from Sigma Aldrich Co. Ltd., Irvine, UK.

### 2.4. Screening for Secondary Metabolites

The aqueous root extract was screened to ascertain the presence of phytochemicals using standard procedures described elsewhere [8].

### 2.5. Animals

Sprague-Dawley rats of either sex, weighing within 200–250 g, and ICR mice (20–25 g) were obtained from the animal house facility of the Department of Biomedical and Forensic Sciences, University of Cape Coast (UCC). All procedures and techniques used in these studies were in accordance with the National Institute of Health Guidelines for the Care and Use of Laboratory Animals.

Ethical approval was obtained from the University of Cape Coast Review Board and adherence to the ethical protocols was ensured by the Department of Biomedical and Forensic Science Ethics Committee.

### 2.6. Suppressive Test

To evaluate the prophylactic activity of the extract, twenty-five mice were randomly assigned to five groups and each group was daily pretreated orally with 30, 100, or 300 mg/kg of the* H. barteri* (Groups 1–3), SP (Group 4), or 10 mL/kg/day normal saline (Group 5). The drug administration was continued for 3 consecutive days. On the fourth day, all the mice were infected intraperitoneally with 1 × 10^6^ *P. berghei* and, 72 hours later, thick films were prepared from blood drawn from the tails of the mice. The parasite density and percentage chemosuppression for all the treatment groups were determined [9, 10].

### 2.7. Curative Antimalarial Test

To assess the curative potential of* H. barteri* on established* P. berghei* infection, twenty-five mice were individually infected with 1 × 10^6^ *P. berghei* and assigned to five groups and monitored for parasite multiplication and establishment. After three days, the animals were daily treated orally with 30, 100, and 300 mg/kg extract (Groups 1–3), 1.14 : 6.9 mg/kg/day A-L (Group 4), and 10 mL/kg normal saline daily (Group 5) for 5 days. The parasite density was monitored daily for five days as described elsewhere [9].

### 2.8. Histopathological Examination

At the end of the observational period, two animals from each treatment group were sacrificed under ether anesthesia and liver, spleen, and kidney were harvested from all animals for gross necropsy and histopathological examination. The organs were placed into fixative (10% formaldehyde) immediately after collection. The tissues were embedded in paraffin; 8 *μ*m sections were cut on a microtome (Bright 5040, Bright Instrument Company Ltd., England) and processed for routine haematoxylin-eosin staining. Slides of tissue sections were observed using trinocular clinical light microscope with a digital camera (Olympus CX1, Japan) connected to a computer. Micrographs of the tissue were generated using the ×10 objective lens for further analysis.

### 2.9. Carrageenan-Induced Rat Paw Oedema

Paw edema was induced in Sprague-Dawley rats with of 100 *μ*L of 1% carrageenan in the left hind foot pad. Paw diameters were determined using a digital calliper. After 2.5 h, rats were treated orally with different doses of* H. barteri* aqueous extract (30, 100, and 300 mg/kg), 10 mg/kg of diclofenac sodium, and normal saline. The paw diameter was measured hourly for three hours after the various treatments [11].

### 2.10. Thermal Nociception

The tail immersion test was used to determine the effect of the extract on nociception. The distal portion of the tail (3-4 cm) of the rat was immersed in hot water maintained at 52°C temperature until the tail was withdrawn. The duration of immersion was recorded and a cut-off time of 10 s was used. The rats were treated with 30, 100, and 300 mg/kg extract and nociception was measured hourly for four hours [12].

### 2.11. Data Analysis

Data were analyzed with GraphPad Prism Version 5 (GraphPad Software, San Diego, CA, USA). The results are presented as mean ± SEM. To compare differences between groups for the antimalarial and anti-inflammatory study, one-way ANOVA was performed with* post hoc* Newman-Keuls' test. The areas under the curves (AUCs) were calculated from behavioural data (time course curves) obtained in the thermal nociceptive study and analyzed using two-way ANOVA followed by* post hoc* Bonferroni's test. *P* < 0.05 was considered statistically significant for all tests.

## 3. Results

### 3.1. Phytochemical Screening of the Aqueous Root Extract of* Haematostaphis barteri*

The aqueous root extract contained triterpenoids, saponins, and glycosides. Alkaloids, anthraquinones, flavonoids, steroids, and tannins were however absent.

### 3.2. Antimalarial Evaluation of* H. barteri *Root Extract

**Figure 1:**
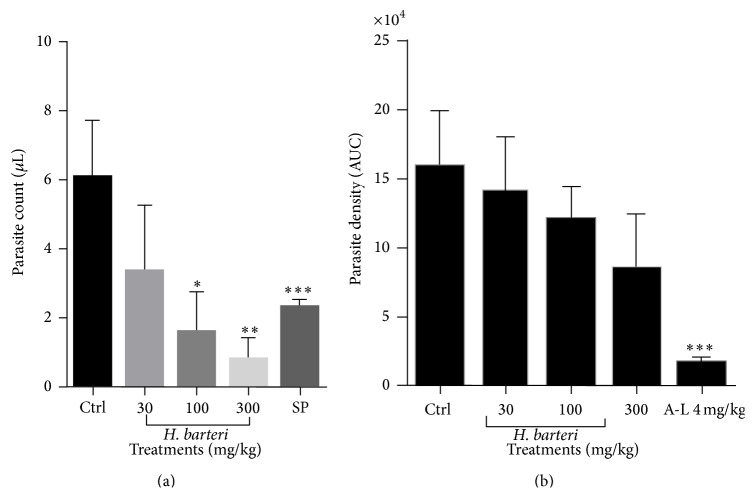
The effect of* H. barteri* root extract presented as area under the curve (AUC) from the time course curves in (a) the prophylactic and (b) curative malaria tests. Data is presented as mean ± SEM. ^*∗∗∗*^ *P* < 0.001, ^*∗∗*^ *P* < 0.01, and ^*∗*^ *P* < 0.05 compared to vehicle-treated group (one-way analysis of variance followed by Tukey's* post hoc* test).

The aqueous root extract of* H. barteri* significantly and dose-dependently reduced the parasite density in the prophylactic test similar to the sulphadoxine-pyrimethamine (Figure 1(a)). In the curative test, however,* H. barteri* decreased the parasite density dose-dependently but this effect was not significant when compared to the control (Figure 1(b)). The standard drug used, artemether/lumefantrine, significantly (*P* < 0.001) reduced the parasite density.

### 3.3. Histological Examination of Organs

Histological studies revealed some damage caused by the malaria parasite and extent of reduction of this damage by the administered 30–300 mg/kg of extracts. The accumulation of iron in the liver and spleen (haemosiderosis), death of cells of the liver (hepatic necrosis), and marked damage of the nephron in the kidney (tubular nephrosis) were to some extent reversed by the extracts. All this damage was severe in the negative control group.

**Figure 2:**
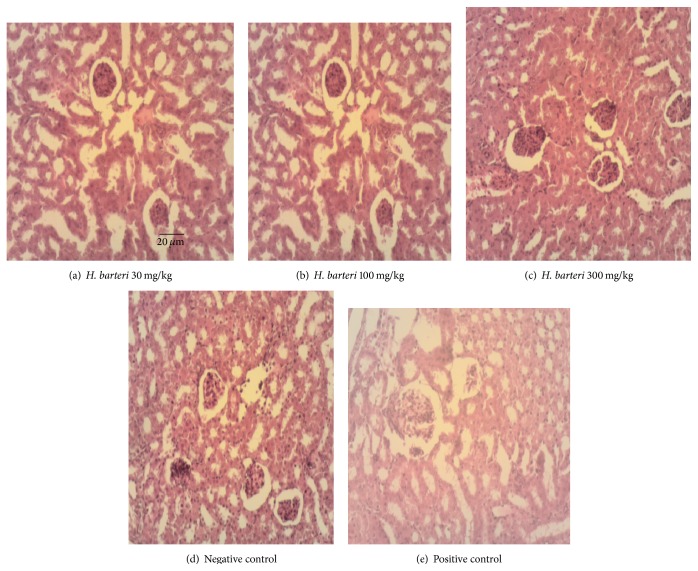
Pictures of kidneys under high magnification (100x). (a)* H. barteri* (30 mg/kg), extract treated kidney: mildly reduced tubular nephrosis; (b)* H. barteri* (100 mg/kg), extract treated kidney: mild pathological changes and renal cortical congestion; (c)* H. barteri* (300 mg/kg), extract treated kidney: slightly edematous, wider capsular space and few basophilic cells; (d) negative control kidney: severe tubular nephrosis and perivascular interstitial mononuclear cell infiltration; (e) positive control (artemether/lumefantrine) treated kidney: mild pathological changes.

**Figure 3:**
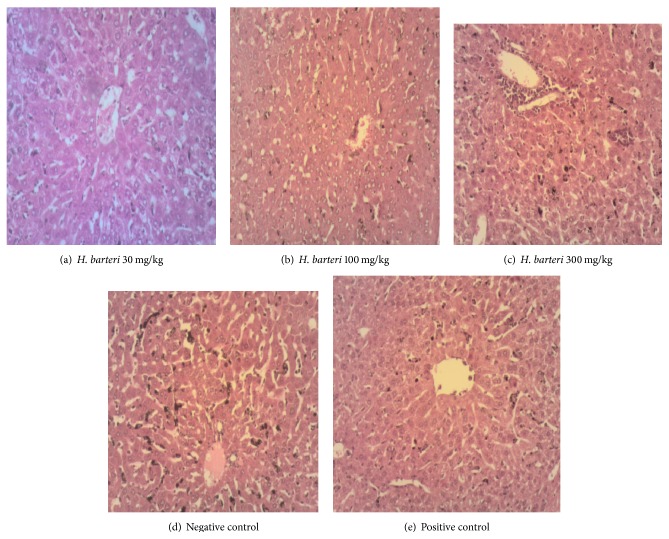
Sections of liver under high magnification (100x). (a)* H. barteri* (30 mg/kg), extract treated liver: few hepatocytes contain merosome, and central vein and portal triad appear normal; (b)* H. barteri *(100 mg/kg), extract treated liver: hepatic necrosis and Kupffer cell hyperplasia; (c)* H. barteri *(300 mg/kg), extract treated liver: mild haemosiderosis, Kupffer cell hyperplasia, and hepatic necrosis, with traces of periportal mononuclear cells infiltration; (d) liver of negative control mouse: severe hepatic necrosis with Kupffer cells hyperplasia and haemosiderosis; (e) artemether/lumefantrine treated liver: mild hepatic necrosis and traces of periportal mononuclear cells infiltration.

**Figure 4:**
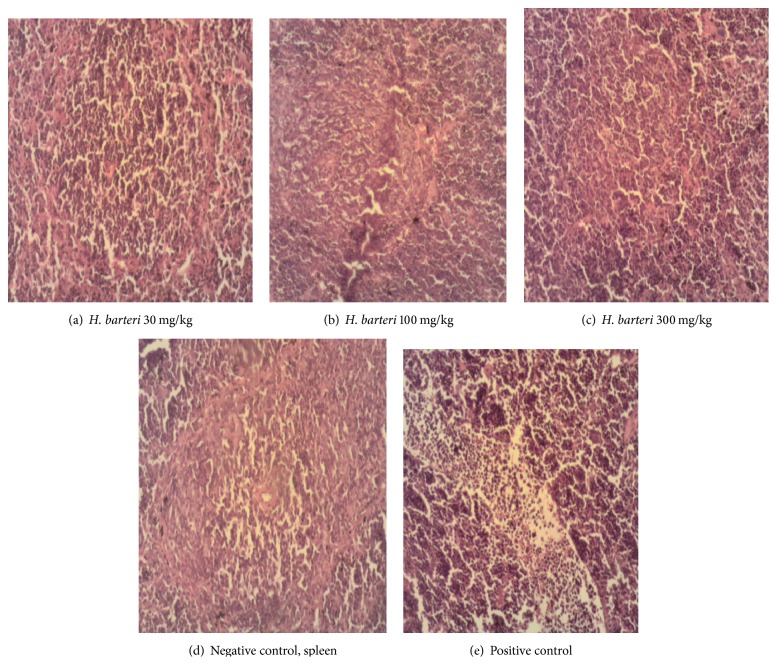
Sections of spleen under high magnification (100x). (a)* H. barteri* (30 mg/kg), extract treated spleen: mild reduction of these pathologies caused by the parasite in the tissues; (b)* H. barteri *(100 mg/kg), extract treated spleen: mild haemosiderosis and loss of cellular architecture; (c)* H. barteri *(300 mg/kg), extract treated spleen: mild haemosiderosis and lymphoid hypoplasia; (d) spleen of negative control: severe haemosiderosis; (e) artemether/lumefantrine treated spleen: mild haemosiderosis and severe megakaryoblast hyperplasia.

There was absence of Kupffer cell hyperplasia and haemosiderosis in the artemether/lumefantrine treated group, but mild hepatic necrosis was still observed (Figure 3). The extract, on the other hand, mildly reduced Kupffer cell hyperplasia, haemosiderosis, and hepatic necrosis (Figure 2(c)). In the kidney, artemether/lumefantrine prevented the infiltration of perivascular interstitial mononuclear cells which are located between tubules of the nephron (Figure 2(e)). An ameliorative effect was observed for the 30 extract treated rats (Figure 3(a)). Lymphoid hypoplasia was observed in the spleen of mice treated with* H. barteri* and the artemether/lumefantrine group (Figure 4). These histological changes were not observed in the negative control group (Figure 4(d)). In all the organs assessed, except for the liver, infiltration of interstitial mononuclear cells was not observed in mice treated with the standard drug and 30 mg/kg extract.

### 3.4. Anti-Inflammatory and Antinociceptive Property of* H. barteri*

**Figure 5:**
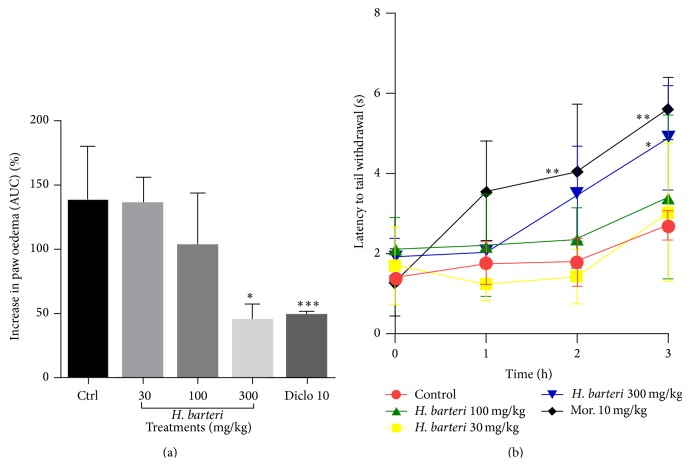
The effect of* H. barteri* root extract on (a) carrageenan-induced inflammation (AUC) and (b) tail withdrawal in the thermal nociception test (time course curve). Data are presented as mean ± SEM. ^*∗∗∗*^ *P* < 0.001 and ^*∗*^ *P* < 0.05 compared to vehicle-treated group (one-way analysis of variance followed by Tukey's* post hoc* test).

The extract at the highest dose administered ameliorated the inflammation induced with carrageenan (Figure 5(a)). The 30, 100, and 300 mg/kg reduced inflammation by 2.9%, 25.9%, and 64.9%, respectively (Figure 5(a)). The extract increased the tail withdrawal latency indicative of antinociception (Figure 5(b)).

## 4. Discussion

Malaria manifests symptoms of pyrexia, bodily pains underpinned by inflammation. Malaria parasite,* P. berghei* infection in laboratory mice, has proven to be very valuable model for detecting antimalarial agents. The ideal antimalarial agent should possess antiplasmodial, anti-inflammatory, antipyretic, and nociceptive activities.

The preliminary investigation into the potential antiplasmodial, anti-inflammatory, and analgesic property of the root extract of* H. barteri* has indicated that the plant has the potential as an antimalarial agent. The leaves and stem extracts have also been reported to exhibit antiplasmodial, anti-inflammatory, and analgesic property [9, 13]. The root extract of* H. barteri *was effective as a prophylactic agent but not a curative antimalarial candidate. The prophylactic activity of the root extract could be attributed to the phytochemicals such as saponin, glycosides, and triterpenoids. These phytochemicals have been reported to exhibit antiplasmodial activity via several mechanisms [14, 15]. The prophylactic activity exhibited by the root extract of* H. barteri* could be attributed to the inhibition of the cytokines production and other mechanisms induced by the parasite in the host for survival [14].

Though the curative activity of the root extract was not significant as compared to A-L (positive control), the possible explanation is that the parasite proteins exported to the bloodstream might have interfered with the phytochemicals thereby reducing their binding properties. Although the pharmacokinetic study has not been done, it is also possible that the phytochemicals might have been metabolized by the mice resulting in sublethal concentration in the bloodstream which could only exhibit prophylactic activity but not a curative one. The curative activity of the root extract was also corroborated by the result of the extract in the anti-inflammatory test where the efficacy of the extract was only significant at the highest dose used. Glycosides, saponins, and triterpenoids have been reported to inhibit inflammation by suppressing the actions of TNF-alpha, interferon gamma, PGE_2_, iNOS, and NF-*κ*B [16–19].

The mode of action of the root extract of* H. barteri* is not known but it is palpable that its ability to restore the proinflammatory and anti-inflammatory cytokines ratio during malaria infection is insufficient at the lowest dose employed in the curative studies [8].

Despite the widespread use of medicinal plants in the treatment of diseases, few scientific studies have been undertaken to ascertain the safety and efficacy of herbal products in disease states. The study of the safety of medicinal plants in disease states is useful because the clearance of these herbal products from the body may vary from a healthy body system to a diseased one.

The severe damage observed in the liver of the negative control group was as a result of parasite life cycle during erythrocytic stages in the blood stream and toxins produced by the parasite. In response to the damage caused, macrophages (Kupffer cells) in the liver actively proliferate (Kupffer cell hyperplasia) breaking down ruptured red blood cells by phagocytic action and splitting the haemoglobin molecules. This results into pathological effects like accumulation of iron in the liver (haemosiderosis), which is usually linked to anaemia and could sometimes lead to liver cirrhosis accompanied by extensive and rapid death of parenchyma cells of the liver (hepatic necrosis). Although haemosiderosis and hemozoin were observed in the liver of the extract treated groups, the levels were not as elevated as that seen in the negative control group. This demonstrates the ability of the extract to reduce the toxicity of malaria infection on the liver.

In the kidney, artemether/lumefantrine prevented the infiltration of perivascular interstitial mononuclear cells which are located between tubules of the nephron. This may be due to the activities of these drugs to mildly reduce destruction of the nephron (tubular nephrosis) by the malaria parasites.

Similarly, haemosiderosis was also observed in the spleen. This organ is the site for the breakdown of worn-out red blood cells and stores the iron they contain. The spleen is also known for pitting hence the parasite and the products are harvested by this organ. Therefore, the spleen also contributed to the accumulation of these iron molecules which was also noticed in the liver of the group treated with artemether/lumefantrine. Atrophy of spleen seen in treated rats will likely be an indication of the potential toxicity of these antimalarial drug (A-L) and candidate (extract). This is because these histological changes were not observed in the negative control group. However, it is clear that the lowest dose of the extract was fairly nontoxic in all the organs assessed. The extract exhibited anti-inflammatory and analgesic properties only at the highest dose. The carrageenan-induced inflammation is a classical model of oedema formation and hyperalgesia, which has been widely used to investigate nonsteroidal anti-inflammatory drugs and analgesics [20–22]. This is not surprising as the root extract of* H. barteri* revealed the presence of triterpenoids, saponins, and glycosides. These secondary metabolites have been reported to attenuate inflammatory diseases such as malaria.

## 5. Conclusion

In conclusion, the root extract of* H. barteri* has demonstrated analgesic, anti-inflammatory, and prophylactic antimalarial activities against* P. berghei*-induced malaria and it is potentially devoid of curative action as per the doses employed in this study. The analgesic, anti-inflammatory, and prophylactic actions against* P. berghei *infection may be attributed to, among other things, secondary metabolites such as glycosides, saponins, and triterpenoids. The extract may have potential toxic effect on people suffering from malaria at the middle and highest doses used.
